# Protocol to quantify mechanical properties of cells using optical tweezers

**DOI:** 10.1016/j.xpro.2025.104189

**Published:** 2025-11-11

**Authors:** Wessel S. Rodenburg, Jorine M. Eeftens

**Affiliations:** 1 Institute for Molecules and Materials, Radboud University, Heyendaalseweg 135, 6523 AJ Nijmegen, the Netherlands; 2 Radboud Institute for Molecular Life Sciences, Radboud University, Geert Grooteplein-Zuid 28, 6525 GA Nijmegen, the Netherlands

**Keywords:** Biophysics, Single-molecule assays, Cell biology, Single cell

## Abstract

Mechanical properties of cells determine their ability to deform when subjected to force, which is crucial in many biological processes. Here, we present a protocol to quantify these properties on living cells by applying piconewton-range forces with optically trapped microspheres. We describe steps for sample preparation of both adherent and suspended cells, calibration of optical trap stiffness, and generation of force-deformation curves. We next detail how to quantify cellular mechanical properties from the resulting data.

For complete details on the use and execution of this protocol, please refer to Rodenburg et al.^1^

## Highlights

• Steps to measure cell mechanics with optical tweezers
• Guidance for sample preparation and instrument setup
• Instructions for quantification and analysis of mechanical properties

## Abstract (editor highlights)

Publisher’s note: Undertaking any experimental protocol requires adherence to local institutional guidelines for laboratory safety and ethics.

## Graphical abstract

**Figure:**
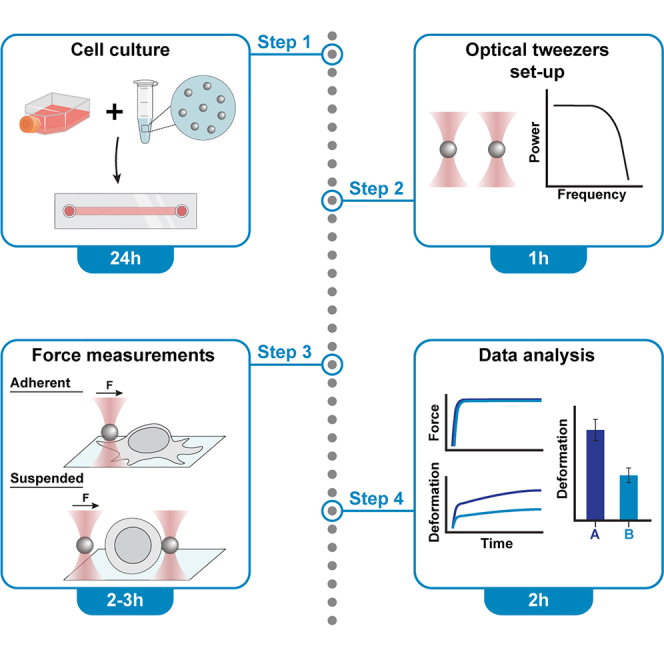

## Before you begin

This protocol includes the specific steps to measure mechanical properties of both adherent and suspended cells. For demonstration purposes, this protocol uses HEK293T cells. We have successfully used this protocol to quantify mechanical properties of other cell lines, including HeLa and B16F10 cells. Small adaptations in sample preparation might be necessary depending on the cell type.

Confirm the flow cell you are preparing your cells in is compatible with your optical tweezers instrument. We recommend using a flow cell rather than a standard cell culture dish, as it allows for efficient exchange of medium and buffers and requires small sample volumes. Adaptations in volumes might be necessary depending on your flow cell of choice.

This protocol requires an optical tweezers setup equipped with a force-feedback system: the ability to maintain a constant force. This enables precise manipulation and measurement of the mechanical response. In addition, a controllable stage in X-, Y- and Z-directions is required to navigate within the flow cell. For measuring on adherent cells, a single optical trap is sufficient, but suspended cells require two optical traps. We use a commercially available dual-trap optical tweezers system (C-Trap, LUMICKS), including software to control the system (Bluelake, LUMICKS). This protocol is formulated in a general manner, so users of other commercial instruments and homebuilt set-ups will also benefit.

### Innovation

Here, we describe a protocol to apply precise pN-range forces to cells using optical tweezers. This protocol is applicable to various cell types. Previous work, often limited to red blood cells, used a stretching approach, where two optically-trapped beads are adhered to either side, and one optical trap is then displaced to stretch the cell.^2^^,^^3^^,^^4^^,^^5^^,^^6^ In contrast, our protocol focuses on indentation of cells using optically-trapped polystyrene beads. This enables measurements on cells that are adherent on a surface (using a single optical trap) and cells in suspension (using two optical traps). To maintain consistency across measurements, the optical trap is under control of a force-feedback system, ensuring that each cell experiences the same force. This robust approach allows for quantification of deformability, spring constant, and creep response of a cell, i.e., how the cell deforms under a constant force.

### Institutional permissions

All work using live human cells was conducted according to institutional and national regulatory standards. Readers will need to acquire permissions from the relevant institutions prior to using this protocol.

### Preparing cells

Cell culture should be started at least one week prior to performing this protocol, allowing cells (in this protocol, HEK293T cells) to recover and ensuring that they are actively dividing and healthy. Cells should be passaged upon reaching a confluence of ∼70–80%. In the steps below we describe the general steps for passaging HEK293T cells in T25 flasks. Volumes should be adapted accordingly when using other types of cell culture dishes or flasks.

**CRITICAL:** All cell culture work must be performed under sterile conditions in a biological safety cabinet, and is performed according to local safety regulations.

1. Pre-warm growth medium (GM) and trypsin-EDTA in a 37°C water bath.
2. Aspirate medium from the cell culture flask or dish.
3. Wash cells once with PBS (add a few mL, such that the entire area is covered).
4. Aspirate PBS and add 0.5 mL trypsin-EDTA. Incubate for 5 min in a 37°C incubator.
5. Inactivate trypsin by adding 4.5 mL of GM. Pipette up and down a few times to ensure all cells have detached from the surface.
6. Transfer the 5 mL of cells into a 15 mL falcon tube. Spin down for 5 min at 500 × *g*.
7. Aspirate medium and resuspend the cell pellet in 5 mL GM.
8. Add 4.5 mL GM in a new flask.
9. Transfer 0.5 mL cells (1:10 dilution) into the new flask. Using this dilution, the cells will typically need to be passaged again in 2 days.

## Key resources table

**Table:** 

REAGENT or RESOURCE	SOURCE	IDENTIFIER
**Chemicals, peptides, and recombinant proteins**		

DMEM, GlutaMAX supplement	Thermo Fisher Scientific	10566016
Phosphate-buffered saline, 1×	N/A	N/A
Trypsin-EDTA (0.05%), phenol red	Thermo Fisher Scientific	25300054
Penicillin-streptomycin (100×)	Thermo Fisher Scientific	10378016
Fetal bovine serum	Biowest	S1400-500
HEPES (1 M)	Thermo Fisher Scientific	15630080
Fibronectin	Sigma-Aldrich	341631

**Experimental models: Cell lines**		

HEK293T	ATCC	N/A

**Software and algorithms**		

Pylake	LUMICKS	https://doi.org/10.5281/zenodo.4280788
Bluelake	LUMICKS	N/A
SciPy	N/A	https://scipy.org/

**Other**		

Polystyrene particles, 3.0–3.4 μm	Spherotech	PP-30-10
μ-Slide I Luer, 0.4 mm, uncoated	ibidi	80171
C-Trap	LUMICKS	N/A

## Materials and equipment

• Prepare cell culture medium: DMEM GlutaMAX supplemented with 10% fetal bovine serum and 1% penicillin-streptomycin. Mix well and store at 4°C for up to two months. From here on, this medium will be referred to as growth medium (GM).
• Prepare a separate solution of cell culture medium supplemented with 25 mM HEPES by adding 1 mL of 1 M HEPES to 39 mL of the medium prepared in step 1. Store at 4°C for up to two months. From here on, this medium will be referred to as live cell medium (LCM).
• Prepare the bead solution by diluting the stock solution polystyrene beads (5% w/v, 3.15 μm average diameter) 1:200 in sterile PBS. Store at 4°C for up to a year.
• Aliquot fibronectin at a concentration of 1 mg/mL in sterile PBS. Store at −20°C for up to a year.

## Step-by-step method details

### Sample preparation for adherent cell measurements

**Timing: 24 h**

This part of the protocol comprises the sample preparation specifically for optical tweezers measurements performed on adherent cells. Cells are seeded in a fibronectin-coated flow cell and incubated overnight to allow firm attachment to the surface. Execute this part of the protocol in a biological safety cabinet.

1. Dilute fibronectin to 10 μg/ml in sterile 1× PBS and add directly into the flow cell channel, such that the entire area is covered (approximately 110 μL is sufficient). Incubate for 2 h at room temperature (RT).

***Note:*** Place the pipette tip directly in the flow cell channel. Slightly tilting the flow cell while pipetting aids the flow of liquid into the channel.

***Note:*** Other adhesive proteins, such as poly-l-lysine, may also be used for flow cell coating. Alternatively, commercially available pre-coated flow cells may also be used.

2. Remove fibronectin from the flow cell channel by filling one reservoir with growth medium (GM), slightly tilting the flow cell, and removing excess liquid from the opposite reservoir.
  a. Repeat three times.
  b. Remove excess liquid from the reservoirs.

***Note:*** Aspirating is not recommended, as it may introduce air bubbles into the flow cell channel.

3. Harvest cells in pre-warmed GM (see “preparing cells”) and determine the cell concentration with an automatic cell counter or hemocytometer.

***Optional:*** In this step, cell viability can be checked using trypan-blue staining.

**CRITICAL:** Cells should be harvested at a confluency of 60–80%.

4. Dilute a total of 30,000 cells in 200 μL GM. Add 60 μL of cells in one reservoir, and remove 60 μL from the opposite reservoir.
  a. Repeat three times.
  b. Remove excess liquid from the reservoirs.
5. Check whether single cells are well dispersed under a microscope.
6. Fill the reservoirs with GM, close the reservoirs with caps, and incubate at 37°C and 5% CO_2_ for 24 h.
7. Empty excess medium from the reservoirs.
8. Add beads and prepare flow cell.
  a. Vortex the bead solution.
  b. Dilute 3 μL of bead solution (see “materials and equipment”) in 197 μL of live cell medium (LCM). Add 60 μL of this solution in one reservoir, and remove 60 μL from the opposite reservoir. Repeat three times.
  c. Fill the reservoirs with LCM.
  d. Close the reservoirs with caps.

***Note:*** HEPES is included to maintain pH stability and compensate for the absence of CO_2_ outside of the incubator. Modify these conditions as needed to suit the specific requirements of your cell type.

**CRITICAL:** Pipette carefully to prevent cell detachment.

9. Flow cell is ready for measurements.

### Sample preparation for suspended cell measurements

**Timing: 2 h**

This part of the protocol comprises the sample preparation for optical tweezers measurements performed on suspended cells. Cells are kept in the suspended state in an uncoated flow cell. Execute this part of the protocol in a biological safety cabinet.

10. Harvest cells in pre-warmed LCM (see “preparing cells”) and determine the cell concentration with an automatic cell counter or hemocytometer.

***Optional:*** In this step, cell viability can be checked using trypan-blue staining.

**CRITICAL:** Cells should be harvested at a confluency of 60–80%.

11. Add beads and prepare flow cell.
  a. Vortex the bead solution.
  b. Dilute 3000 cells and 3 μL of bead solution (see “materials and equipment”) in a total volume of 110 μL of LCM.
  c. Add directly inside the flow cell channel, such that the entire area is covered.

***Note:*** Place the pipette tip directly in the flow cell channel. Slightly tilting the flow cell while pipetting aids the flow of liquid into the channel.

***Note:*** HEPES is included to maintain pH stability and compensate for the absence of CO_2_ outside of the incubator. Modify these conditions as needed to suit the specific requirements of your cell type.

12. Check whether single cells are well dispersed under a microscope.
13. Fill the reservoirs with LCM, close the reservoirs with caps, and incubate for 30 min at 37°C and 5% CO_2_ to allow the cells to settle.
14. Flow cell is ready for measurements.

### Optical tweezer setup and calibration

**Timing: 1 h**

In the steps described below, beads are optically trapped and the optical trap(s) are calibrated to obtain the trap stiffness (*ĸ*). The trap stiffness (*ĸ*) is used to convert a displacement of the bead from the optical trap center (*Δx*) into force (*F*) according to Hooke’s law: *F* = *κ*∗Δ*x*. Correct acquisition of the trap stiffness is thus essential for accurate force readout. Various methods have been developed to calibrate optical tweezers, each with advantages and limitations (reviewed in^7^), and the best calibration protocol may depend on the optical tweezers instrument used. Here, for the LUMICKS C-Trap, a calibration method is used based on the power spectrum of bead motion within an optical trap.^8^^,^^9^^,^^10^

15. Position the flow cell in a sample holder appropriate for your specific optical tweezers setup. Adjust the objective and condenser until the beads are visible in the bright-field view.

***Note:*** While the cells are present at the bottom surface of the flow cell, the beads may reside at a slightly higher Z-position.

16. Turn on the trapping laser(s) at relatively low laser power (∼1/3 of the laser power used for measurements). Trap one bead (for measurements on adherent cells) or two beads (for measurements on suspended cells).

***Note:*** To trap a bead, the laser should be positioned in the vicinity of a bead, either by adjusting the position of the stage, or the lasers themselves. When a bead is near the laser, it will be trapped into optical trap center. When trapped, the bead position(s) can be controlled by moving the laser.

***Note:*** If beads do not get trapped easily into the optical trap, you may increase the laser power. However, to minimize potential damage to cells, it is best practice to use the lowest laser power necessary for effective trapping and manipulation.

***Note:*** Over time, some beads may start to stick to the bottom surface of the flow cell. These beads are difficult to trap – it is therefore advised to trap ‘free’ floating beads.

17. Move the stage along its Z-direction towards the bottom surface of the flow cell, until cells (either adherent or suspended) are in focus. Navigate the trapped bead(s) toward an area where no cells are in the vicinity.

**CRITICAL:** Cells in the vicinity of the trapped beads may influence local fluid flow, and, thus, the calibration of the beads. Calibration should therefore be carried out in the absence of cells.

**CRITICAL:** Hydrodynamic drag from proximity to the bottom of the flow cell can influence force measurements. Aim to perform calibration and experiments at sufficient distance from the surface (∼5 μm) to minimize this effect.

18. When using bead-tracking software, make sure that the beads are properly recognized by the software.
  ***Note:*** The bead-tracking software (LUMICKS) that we use, requires a template to track the position of the beads.
  a. Carefully draw the bead-tracking ROI around the bead.
  b. Enter the average diameter of the beads as provided by the manufacturer.
  c. Check whether the beads are properly recognized (indicated by a percentage).
19. Increase the laser power to the same power that will be used for the measurements.
20. Perform the calibration. To accurately obtain the power spectrum, the motion of the bead within the optical trap is recorded for 10 s (for more details refer to ref.^8^^,^^9^^,^^10^). Aim for a trap stiffness around 0.20–0.25 pN/nm.

**CRITICAL:** When using both optical traps (for measurements on suspended cells), ensure that the beads are aligned horizontally with at least a few μm distance in between them.

**CRITICAL:** If any debris is dragged into the optical trap during calibration, release the bead, trap another and redo the calibration.

21. When using both optical traps (for measurements on suspended cells), we typically regard deviations between the stiffness of the two traps within 10% as acceptable. If deviations are larger, release the beads and redo the calibration.

***Note:*** Large deviations in trap stiffness may have several sources, for instance a difference in bead size or presence of debris in the optical trap, and should therefore be prevented.

### Force application to adherent and suspended cells

**Timing: 2–3 h**

This part of the protocol describes the steps to apply force to adherent and suspended cells by means of a force-feedback system. This system calculates deviations from a pre-defined force (‘target force’) at a certain frequency and adjusts the position of the optical trap accordingly, ensuring that a constant force is applied. The position of the bead(s) is simultaneously tracked, from which the deformation of the cell can be inferred. This way, one can quantify how the cell deforms under a constant force, and ensure force application is consistent across cells.

22. Navigate the trapped bead(s) toward a cell by moving the stage and/or the lasers. Aim to have the Z-position of the beads roughly aligned with the middle of the cell, using the bright-field view as a guide.

**CRITICAL:** The Z-position of the optical trap within the flow cell may influence the trap stiffness. Recalibrate if large adjustments in the Z-position of the beads are required.

**Figure 1:**
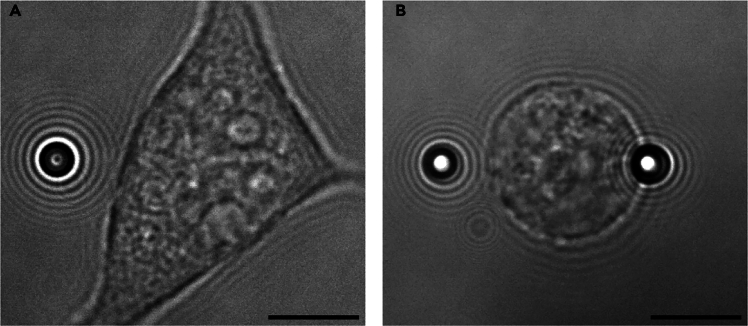
Starting positions of the optically-trapped beads prior to force application Example bright-field images of (A) an adherent cell, and (B) a suspended cell. Optically-trapped polystyrene beads are positioned in the ‘starting positions’ prior to starting the force-feedback measurement. For measurements on adherent cells, only one bead is used to apply force. For measurements on suspended cells, one bead (left) is used to apply force, while the other bead (right) is used to stabilize the position of the cell. Scale bar = 10 μm.

***Note:*** Monitoring the force measured by trap 2 while moving this trap towards the cell aids in recognizing when the bead contacts the cell. A sudden peak in force of several pN is indicative of contact.

23. Position the bead(s) to the starting positions as depicted in Figure 1.
  a. The bead in trap 1 (left) should be positioned a few μm away from the cell.
  b. For measurements on suspended cells (Figure 1B), the bead in trap 2 (right) is moved toward the cell until contact is made. The bead in trap 1 applies force, while the bead in trap 2 serves to stabilize the position of the suspended cell.

**CRITICAL:** For measurements on suspended cells, it is important that the beads are aligned horizontally as in Figure 1B.

24. When performing this protocol for the first time, it is important to first test and optimize the force-feedback parameters. The specific parameters likely differ per optical tweezers setup, but typically requires the user to define the following:
  a. **Target force.** The maximum target force that can be applied depends, among other things, on the trap stiffness, but is typically no more than 400 pN.^11^ We previously applied constant forces of 50 and 100 pN.^1^
  b. **Direction.** Trap 1 should move along the lateral (*x*) direction toward the cell.
  c. **Frequency.** The frequency at which deviations from the target force are measured and subsequent corrections in the trap position are executed. A higher frequency will ensure that deviations from the target force are corrected more quickly. It is advised to use the maximum possible feedback frequency. For the LUMICKS C-Trap, the maximum frequency is 31.3 Hz.
  d. **Corrections.** Typically, one or more parameters determine how a deviation from the target force is converted into a command that corrects the trap position. We use a proportional control, that is, an error from the target force is converted into a correction that is proportionally large according to a gain factor *K*_*p*_*.* A high *K*_*p*_ will ensure the target force is reached quickly, but may result in overshooting or oscillations (see troubleshooting). A *K*_*p*_ between 0.5 and 1 typically provides a good balance between maximizing speed and minimizing overshooting.
25. When the beads are positioned as depicted as in Figure 1, check whether the force of trap 1 is zero. Start the force-feedback cycle and apply the target force for at least 10 s, then stop the measurement.
26. The beads typically remain stuck to the cell after the measurement. To measure another cell, release the beads from the traps and trap new beads.

***Note:*** Recalibration is not required when new beads are trapped, as long as critical parameters (e.g. temperature, viscosity, bead size, Z-position, laser power) remain unchanged.

27. For each measurement, export the force and bead position data. For measurements on suspended cells, export the bead position data of both beads.

***Note:*** For C-Trap (LUMICKS) systems, these data are exported as .h5 files.

## Expected outcomes

**Figure 2:**
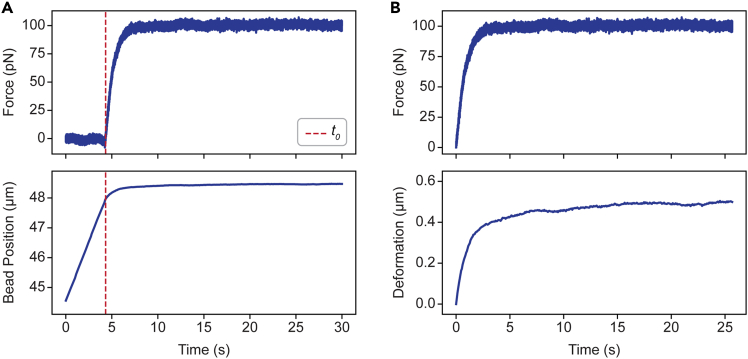
Force-deformation curves (A) Raw force and bead position data of an adherent cell deformed with a target force of 100 pN. Red dashed lines indicate the point of contact (*t*_*0*_). (B) Force and deformation curve of the same measurement as shown in Figure 2A.

For both adherent and suspended cells, the force-deformation curve is expected to consist of (1) an initial elastic deformation and (2) a creep response. A typical example of a force-deformation curve for an adherent cell is shown in Figure 2. For more details, please refer to Rodenburg et al.^1^

## Quantification and statistical analysis

In this step, the analysis is performed to extract cellular mechanical properties (deformation, creep, and spring constant) from the raw data. The analysis is performed using custom-made Python scripts and Pylake.^12^ Here, we show how to generate force-deformation curves and how to calculate mechanical properties using a measurement on an adherent cell as an example.

1. The .h5 files containing raw force and bead position data can be accessed using Pylake.^12^ A typical plot of force and bead position raw data is shown in Figure 2A for an adherent cell deformed with a target force of 100 pN.

***Note:*** This is an example for an adherent cell, in which only one bead is used to deform the cell (Figure 1A). The position of this bead over time is used to extract the deformation of the cell. For measurements on suspended cells, two beads are used (Figure 1B). One should therefore use the distance between the two beads to extract the deformation of suspended cells. For C-Trap (LUMICKS) systems, the distance can be exported directly. Else, subtract the position of bead 1 from the position of bead 2 to derive the distance over time.

**Figure 3:**
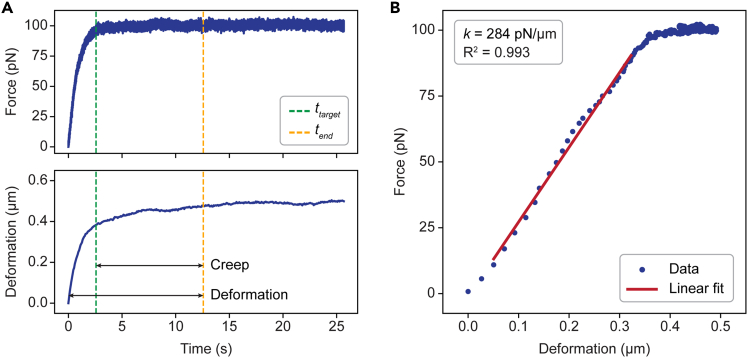
Quantification of the cellular mechanical properties (A) Force and deformation curve (same as in Figure 2). Green dashed lines indicate the time (*t*_*target*_) when the target force is reached. Yellow dashed lines indicate the time (*t*_*end*_) 10 s after *t*_*target*_, used to quantify deformation and creep. (B) Force-deformation curve of the same measurement. Red line indicates the linear fit used to obtain the spring constant (*k*).
5. Quantify deformation: the deformation at 10 s after reaching the target force (*t*_*end*_) (Figure 3A, dashed yellow lines).

***Note:*** You can define *t*_*end*_ depending on your interest.

2. Identify the point of contact. We define the point of contact as the last point before the force increases above 0 pN (*t*_*0*_) (Figure 2A, dashed red lines).
3. Convert the bead position into deformation. This is achieved by subtracting the bead position at the point of contact (*t = t*_*0*_) from the bead position at time *t.* The resulting plot shows cellular deformation over time (Figure 2B).
4. Identify the point where the target force (100 pN) is reached, that is, the time (*t*_*target*_) where force first reaches a value of 100 pN (Figure 3A, dashed green lines).

6. Quantify creep: the deformation under a constant force from *t*_*target*_ until *t*_*end*_ (Figure 3A).
7. To quantify the spring constant (*k*), a direct measure of cellular stiffness, plot force against deformation starting from the point of contact (*t*_*0*_) as shown in Figure 3B.
  a. Use linear regression to fit the data.
  b. The spring constant is the slope of the elastic (linear) part of the force-deformation curve (Figure 3B, red line).

***Note:*** For more accurate fitting, data points in the first and last 10 pN are excluded from the fit.

## Limitations

This protocol enables mechanical characterization of single cells at a rate of ∼10 cells per hour for experienced users. We recommend limiting measurement time to a few hours, as cells become less viable over time, especially in a non-temperature controlled environment.

While optical tweezers enable accurate force application with pN-resolution, the force limit is typically 200-400 pN. These forces are sufficient to accurately measure cell stiffness and deformability.^1^^,^^11^ However, if higher forces are required for an experiment, using optical tweezers is not advised.

This protocol describes the steps to obtain mechanical properties of cells by applying force through indentation. Stretching cells with optical tweezers is also possible but requires careful consideration of experimental set-up. Uncoated beads (as used in this protocol) might detach from the cells before the target force is reached. This can potentially be prevented by bead coatings that can interact with various components of the cell surface. As a result, when applying force through pulling with optical tweezers, the mechanical response may reflect a mix of interactions, complicating interpretation of the data.

## Troubleshooting

### Problem 1

**Figure 4:**
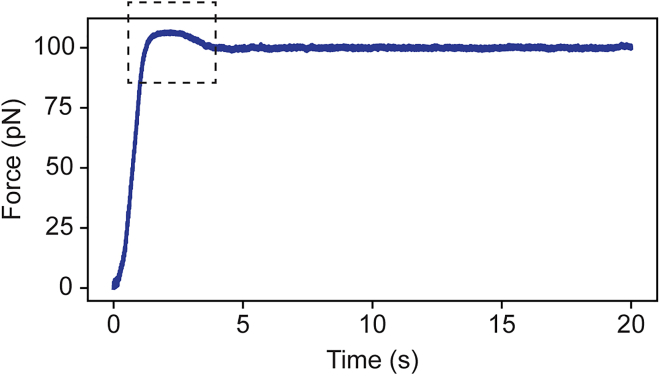
Overshoot of the target force Example curve of a cell deformed with a target force of 100 pN in which a slight overshoot is visible (indicated by the dashed square).

Overshooting (Figure 4) and/or force oscillations when trying to apply a constant force (step 24).

### Potential solution

Adjust the force-feedback parameters. Overshooting or oscillations may arise when deviations from the target force are converted into corrections that are too large. To solve this, the gain factor *K*_*p*_ may need to be lowered. It is advised to start with a *K*_*p*_ of 1. Measure a cell and monitor whether overshooting and/or oscillations are present. If present, lower the value of *K*_*p*_ by 0.1, measure another cell, and check for overshooting and/or oscillations. Repeat until overshooting and/or oscillations are visibly minimized.

### Problem 2

The bead escapes the optical trap during measurements (step 25).

### Potential solution

This problem arises when the bead is ‘pushed’ too far away from the optical trap center, causing it to escape the optical trap. If this occurs frequently, lower the target force.

### Problem 3

The suspended cell does not remain stationary and/or gets dragged toward the optical trap (step 25).

### Potential solution

While adherent cells are firmly attached to the surface, the mobility of cells in suspension can make measurements on suspended cells particularly tricky. First, it may be helpful to incubate the flow cell for a longer time prior to measuring (see “sample preparation for suspended cell measurements”), to ensure that cells have settled and are loosely touching the bottom of the flow cell. Second, lowering the laser power reduces the tendency of the suspended cell to move towards the optical trap.

## Resource availability

### Lead contact

Further information and requests for resources and reagents should be directed to and will be fulfilled by the lead contact, Jorine Eeftens (jorine.eeftens@ru.nl).

### Technical contact

Technical questions on executing this protocol should be directed to and will be answered by the technical contact, Wessel Rodenburg (wessel.rodenburg@ru.nl).

### Materials availability

This study did not generate new reagents or materials.

### Data and code availability

This study did not generate new datasets or code.

## Acknowledgments

We thank members and students of the Eeftens lab for discussion.

## Author contributions

W.S.R. and J.M.E. conceived the project. W.S.R. performed experiments. W.S.R. analyzed data. W.S.R. and J.M.E. wrote the manuscript.

## Declaration of interests

The authors declare no competing interests.
